# Unusual presentation of metastatic adenocarcinoma

**DOI:** 10.1186/1477-7819-5-116

**Published:** 2007-10-18

**Authors:** Izhar N Bagwan, Gary Cook, Satvinder Mudan, Andrew Wotherspoon

**Affiliations:** 1 Department of Histopathology, The Royal Marsden Hospital, Fulham Road, London SW3 6JJ, UK; 2 Department of Nuclear Medicine, The Royal Marsden Hospital, Fulham Road, London SW3 6JJ, UK; 3 Department of Surgery, The Royal Marsden Hospital, Fulham Road, London SW3 6JJ, UK

## Abstract

**Background:**

The most common tumours of the adrenal gland are adenoma, pheochromocytoma, adrenocortical carcinoma, and metastases. Although the imaging features of these tumours are established, the imaging characteristics of uncommon adrenal masses are less well known. In patients with extradrenal tumour, incidental discovery of an adrenal mass necessitates excluding the possibility of metastatic malignancy.

**Case presentation:**

A 52 year-old female was diagnosed with oesophageal adenocarcinoma and treated with oesophagectomy and adjuvant chemotherapy. Sixteen months later on staging CT scan a 2 × 2 cm adrenal mass was detected, which increased in size over a period of time to 3 × 3 cm in size. Adrenalectomy was performed and histological examination revealed metastatic adenocarcinoma within an adrenal adenoma.

**Conclusion:**

The present case highlights the unusual behaviour of an oesophageal adenocarcinoma causing metastasis to an adrenocortical adenoma.

## Background

Due to improvements in radiological diagnosis capabilities, there has been a recent increase in adrenal tumours that are incidentally discovered in patients who underwent medical examination by ultrasonography (US), computed tomography (CT) and magnetic resonance imaging (MRI), i.e. "adrenal incidentalomas" [1]. In patients with extra adrenal tumour, incidental discovery of an adrenal mass necessitates excluding the possibility of metastatic malignancy [2]. Discrimination between benign and malignant adrenal mass lesions is a frequent clinical problem [3]. Important hallmarks used as indications for surgical intervention are the size, growth rate and the imaging characteristics of the tumour as well as the endocrinological behaviour [1]. Here we present an unusual case of metastatic carcinoma within an adrenal adenoma.

## Case presentation

**Figure 1:**
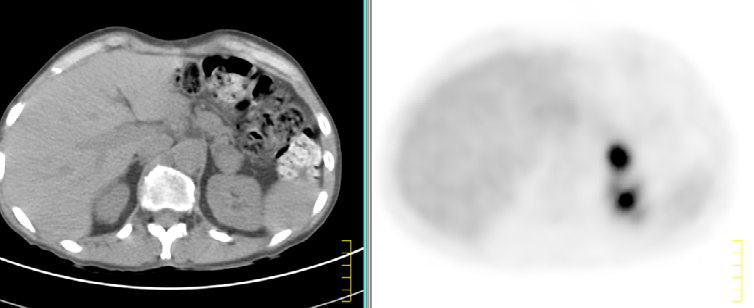
A). CT scan image and B). 18FDG-PET scan.18FDG-PET scan with CT anatomical image fusion (axial view) shows an intense abnormal FDG uptake within the adrenal gland suggestive of metastasis or possibly a primary tumour.

A 52-year-old woman presented with complaints of dysphagia and hoarseness of voice in December 2003. She was diagnosed to have an advanced moderately differentiated oesophageal adenocarcinoma for which adjuvant chemotherapy was started. In May 2004, she underwent a transcervical oesophagectomy and gastric pull up followed by postoperative chemotherapy. The postoperative interval was uneventful. Staging CT scan performed in April 2005 revealed a 2 × 2 cm left adrenal mass. Half body positron emission tomography (PET) scan with 18F-2-fluoro-D-deoxyglucose (FDG) with CT anatomical image fusion performed in April 2005 and again in February 2006 revealed an increasing left adrenal mass measuring 3 × 3 cm in size. The left adrenal gland showed an intense abnormal FDG uptake (Figure 1). This metabolically active lesion was suggested to represent either a metastasis or possibly a primary adrenal tumour. FDG PET scan showed no other foci of metabolically active disease. The endocrine work-up showed that the adrenal tumour was hormonally non-functioning. Left adrenalectomy was performed and the specimen was sent for histopathological evaluation. Preoperative needle biopsy was not performed.

**Figure 2:**
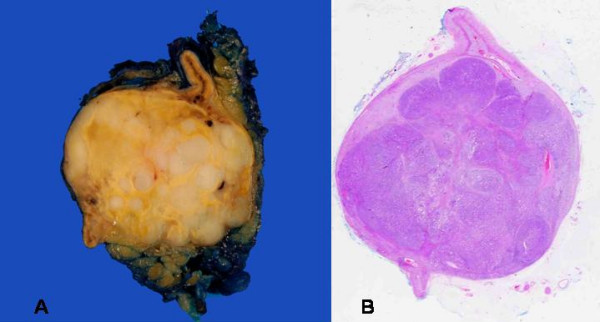
A)-Photograph of the gross specimen of the enlarged adrenal gland showing a mottled spherical lesion measuring 3 cm in diameter, with compressed normal adrenal gland at the periphery. B)-Scanner view showing the well-circumscribed adenoma with foci of metastatic adenocarcinoma. (Haematoxylin and Eosin stain X10)

**Figure 3:**
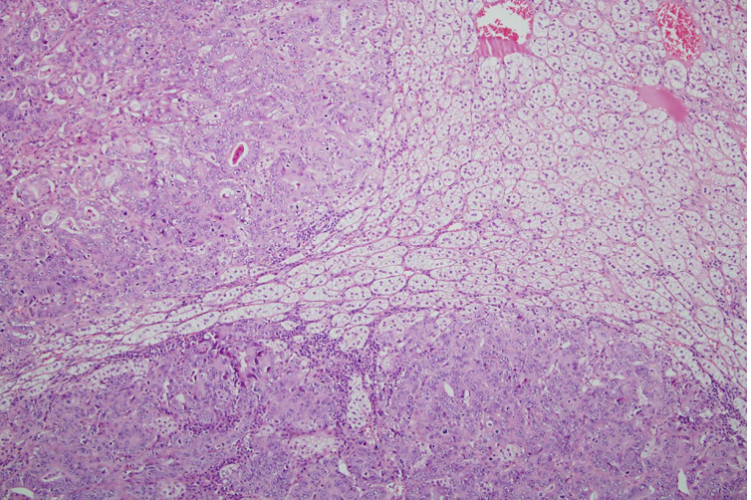
Low power magnification showing an adenoma composed of clear cells arranged in a nesting pattern with metastatic adenocarcinoma. (Haematoxylin and Eosin stain X100)

The adrenal gland measured 6 × 3.5 × 3.0 cm in size and weighed 23 grams. On slicing, the adrenal gland contained a mottled spherical lesion measuring 3 cm in diameter (Figure 2A). Histopathological examination revealed a well circumscribed tumour composed of clear cells arranged in a nesting pattern, separated by thin fibrovascular stroma. Compressed normal adrenal gland parenchyma is seen at the periphery (Figure 2B &3). There was no evidence of necrosis, mitosis or nuclear pleomorphism. The tumour cells stained for Inhibin, Melan A, Calretinin and Neuron Specific Enolase and are negative for Chromogranin, Neurofilament and S100 protein. The features were consistent with an adrenocortical adenoma. In addition, the adenoma showed multiple deposits of moderately differentiated adenocarcinoma (Figure 2B &3). The neoplastic glands stained for AE1/AE3, Cytokeratin 7, Cytokeratin 20 and Carcinoembryonic antigen (CEA). The overall features were consistent with metastasis of moderately differentiated adenocarcinoma, from a known primary in oesophagus, to an adrenocortical adenoma.

The follow-up of the patient was uneventful for 6 months following surgery.

## Discussion

Adrenal mass lesions may represent a variety of pathologic entities, including benign and malignant primary tumours of the adrenal cortex of medulla, secondary malignancies metastatic to the adrenal gland, and other benign conditions such as haemorrhage, granulomatous inflammation and simple cysts. Of these possibilities, the adenomas and metastases make up most of the adrenal mass lesions in the adult population [3]. The adrenal glands are susceptible organs for metastases from various malignancies of which the largest proportion represent spread from lung or breast primaries. Other sites from which adrenal metastases may develop include kidney and ovary and the adrenal may be the site for metastatic melanoma and involvement by lymphoma or leukaemia [1]. An analysis of the tumour registry data (1994–1996) at M.D. Anderson Cancer Centre, revealed that adrenal metastasis occurred in 202 cases out of 4399 cases, with lung and kidney being the primary organs of origin in 51% and 28% of cases respectively. Adrenal metastasis of oesophageal adenocarcinoma was seen in 3% of cases [4]. In a review of autopsy cases, the adrenal glands were involved in 27% of cancer cases and the incidence of adrenal metastases in patients with breast and lung cancer was approximately 39 and 35%, respectively. Among patients with cancer, more than 50% of clinically unapparent adrenal masses are reported as metastases [1]. In the present case, the adrenal mass was removed considering it to be metastatic and the adenomatous component was detected only after pathological examination.

Radiologically, MRI is known to be effective in distinguishing between benign and malignant lesions. Metastases are usually hypointense on T1-weighted images but hyperintense on T2-weighted images. In particular, benign adenomas exhibit clear suppression of the signals on chemical-shift imaging. [1,2]. Unfortunately, chemical-shift MRI was not performed at the initial examination in our present case. Available data regarding the size suggest that lesions smaller than 4 cm are generally benign [5]. But this was not so in our case as the adrenal mass was 3 cm in size. This case demonstrates the advantages of FDG-PET in characterizing adrenal masses in patients with clinically unapparent adrenal masses or cancer work-up including differentiation of malignant from benign adrenal masses. The adrenal glands are not discretely identified on a normal PET scan. However, they are often visible on PET/CT studies with minimal uptake. Focal adrenal uptake is abnormal. But, besides metastatic disease, other causes of focal adrenal uptake include benign and malignant pheochromocytoma, giant adrenal myelolipoma and adrenal carcinoma. These can be differentiated from metastasis by CT or MRI. Although bilateral uptake is worrisome for metastasis, this can also be secondary to adrenal hyperplasia. This should be considered when the bilateral uptake is nodular. Hence it is important to recognize that not all lesions in cancer patients are metastasis [6,7]. The sensitivity and specificity of PET in characterizing adrenal lesions in patients with known malignancy is 100% and 94% respectively. The sensitivity of PET is equal to MRI and superior to CT [6].

## Conclusion

Adrenal gland is a common site of metastasis from various tumours especially of lung or breast, and radiologically the sensitivity of PET in detecting them is equal to MRI and superior to CT. The present case also highlights the unusual behaviour of an oesophageal adenocarcinoma causing metastasis to an adrenocortical adenoma.

## Competing interests

The author(s) declare that they have no competing interests.

## Authors' contributions

IB- wrote the draft manuscript and did the literature search.

GC- performed the radiology and contributed to drafting the manuscript.

SM- performed the surgery and contributed to drafting the manuscript.

AW- performed the pathology, diagnosed the condition and prepared the photomicrograph.

All authors read and approved the final version of the manuscript.

Acknowledgement

The authors are grateful to Lee Gumble for his assistance in photography.
